# Should Preachers Pay?

**Published:** 1887-01

**Authors:** Geo. R. West

**Affiliations:** Rome, Ga.


### SHOULD PREACHERS PAY?

BY GEO. R. WEST, M. D., ROME, GA.

It is the custom all over the country, among medical men, not to charge any fee for professional services rendered a minister of the gospel or his family.

The object of this paper is to protest against this custom:

First. In the interest of the minister.

Second. In the interest of the physician.

1. Such a practice is objectionable on the part of the minister because he cannot have his choice of physicians; he cannot demand attentions that he asks, and cannot so readily change his physician if he becomes dissatisfied. He cannot have his choice of physician. This is true for several reasons. If he sends for the one of his choice, a popular practitioner, at an opportune time, or happens in his office when he is idle, he will receive prompt attention with great courtesy. If, however, he sends when that physician is tired, or if the weather happens to be disagreeable, it being cold, wet or in the night-time, the physician will stay at home when he finds who wants him. If it had not been for that abominable custom which would prevent him from charging for his services, he would have rendered them.

Again, since it is the established custom, the preacher, in his choice of a physician, must consider that the practice will be done as a courtesy, and he must select a member of his church, in preference to a better doctor of some other denomination, as a return of the expected courtesy.

It being understood that the minister is not to put a money value on the physician's services, but simply incurs obligation, if he be a man of ordinary delicacy and politeness, he cannot say to the doctor, "Come to my house this morning," but it is, "Please come up this morning if you are not too busy."

When a doctor's services are wanted they are generally wanted as soon as possible, and if you place the patient beyond the power of demanding immediate attention, you had almost as well place him beyond the power of getting a doctor at all. By accepting for nothing a doctor's services, a preacher places himself in the light of a mendicant and must act accordingly; he appears before the world as a beneficiary, and as such loses the respect due him, from a portion at least, of the people of the community. His salary should be sufficient for him to live upon and pay the usual expenses of living, to compensate his fellow-beings for contact with him, not to be a nucleus that accumulates but never imparts. If his salary is not sufficient for his wants, then he should have, besides his profession of preaching, a vocation that would assist in making a living.

Under the existing customs, if a preacher or his family is being attended by a doctor in whom they lose confidence, it is a very awkward thing to demand that the doctor cease his gratuitous visits and then call in another. If it was a money transaction it could be accomplished in a business way.

2. The custom is a bad one on account of its effect upon the physician.

No doctor should practice his profession for the money that is in it, yet few could afford to practice it if there was no compensation. The original idea may not be that the doctor does not charge the minister because he wants his influence, but that is the practical working of the custom. This, I say, is degrading to the profession. The physician should attend a case, either for charity or money compensation, with a deep interest in the medical points of the case under both circumstances, and I hold that to attend a case for its influence is a pernicious and deceitful practice. It places the insignificant preacher with little influence where he cannot get his physician even if he is willing to pay. It should not be argued that some cannot afford to pay, because in other walks of life men are found who are not able to pay their doctor full prices, yet they are not prevented from demanding his attention with a promise of remuneration in the future. Where a doctor practices for his own pastor, it might be well for him to make this an addition even to his yearly church contribution, but should a doctor be expected to contribute to the support of all pastors any more than any other business man would be? Where the preacher is very poor his account could be reduced in amount, as are those of the poor in general, but let it be done on account of his poverty and not by reason of his being a minister of the gospel.

I recognize the fact that circumstances may make exceptions in country districts. Where there is but one preacher and one doctor in a community, and they are each paid their living in produce, it might be well for the doctor to contribute his services as a part of the preacher's salary, but do not deprive him of the right to give these services by making it the custom, so that the preacher may almost demand them.

As the medical profession has established this unpleasant custom, it lies with them to abrogate the evil.

I have talked with many preachers on this subject and each of them have told me that they have suffered from the evil workings of this absurd custom.

---

TREATMENT OF TETANUS.—Hiller (*Centralblatt für Chirurg. No. 48, 1886*) draws attention to the correspondence of the symptoms of tetanus with those of sunstroke. Both are characterized by an excessive rise of temperature. In fatal cases of tetanus the temperature rises above 104 degrees and just before death it often rises to 109 degrees and 111 degrees. In sunstroke, by comparing the results gained by reliable observers, it was found that the temperature in several cases invariably rose to 108 degrees, even to 112 degrees.

The cause of the high temperature in the two diseases is attributed to increased heat production, caused by great and continued muscular contraction, and decreased heat elimination in tetanus due to tetanic contraction of the cutaneous vessels, in sunstroke to excessive clothing (especially among soldiers) and conditions of the atmosphere. Both diseases have a great mortality as a natural consequence of high temperature.

From the above the author concludes that the treatment should be directed mainly to the reduction of temperature, and such means should be employed that will produce increased heat elimination, such as cold baths, the administration of antipyrin, thallin, pilocarpin, etc.

